# High‐Frequency rTMS Improves Visual Working Memory in Patients With aMCI: A Cognitive Neural Mechanism Study

**DOI:** 10.1111/cns.70301

**Published:** 2025-03-24

**Authors:** Meng Liu, Ren Ren‐Li, Jingnan Sun, Janelle S. Y. Yeo, Jing Ma, Jia‐Xin Yan, BuMaYiLaMu‐XueKeEr, Zhao‐Xi Tu, Yun‐Xia Li

**Affiliations:** ^1^ Department of Neurology Shanghai Pudong Hospital, Fudan University Pudong Medical Center Shanghai China; ^2^ Department of Neurology Shanghai Changhai Hospital, the Second Military Medical University Shanghai, P.R. Shanghai China; ^3^ Department of Neurology Tongji Hospital, School of Medicine, Tongji University Shanghai China; ^4^ Department of Biomedical Engineering Tsinghua University China; ^5^ School of Medicine, University of Sydney Camperdown New South Wales Australia

**Keywords:** alpha oscillations, dorsolateral prefrontal cortex, Fronto‐parietal functional connectivity, repetitive transcranial magnetic stimulation, visual working memory

## Abstract

**Background:**

Visual working memory (VWM), which is an essential component of higher cognitive processes, declines with age and is associated with the progression from amnestic mild cognitive impairment (aMCI) to Alzheimer's disease (AD). Cognitive impairment, particularly in VWM, is prominent in aMCI and may indicate disease progression. This study investigates the cognitive neural mechanisms responsible for VWM impairment in aMCI, with a focus on identifying the VWM processing stages affected. The study targets the dorsolateral prefrontal cortex (DLPFC) for repetitive transcranial magnetic stimulation (rTMS) to investigate its influence on VWM in aMCI patients. The role of the DLPFC in the top‐down control of VWM processing is central to understanding rTMS effects on the stages of information processing in aMCI‐related VWM impairments.

**Methods:**

A 7‐day rTMS intervention was performed in 25 aMCI patients and 15 healthy elderly controls to investigate its effects on VWM and cognitive functions. Tasks included VWM change detection, digital symbol transformation, and the Stroop task for attention and executive functions. EEG analyses consisting of ERP, ERSP, and functional connectivity (wPLI) were integrated. The first part of the study addressed the cognitive neural mechanism of VWM impairment in aMCI and differentiated the processing stages using EEG. The second part investigated the effects of rTMS on EEG processing at different VWM stages and revealed cognitive neural mechanisms that improve visual working memory in aMCI.

**Results:**

The results indicated a significant deterioration of VWM tasks in aMCI, especially in accuracy and memory capacity, with prolonged reaction time and increased duration of the Stroop task. In the VWM memory encoding phase, N2pc amplitude, α‐oscillation in the parieto‐occipital region, and θ‐band synchronization in the frontoparietal connectivity decreased. Conversely, rTMS improved N2pc amplitude, α‐oscillation, and θ‐band synchronization, which correlated with improved frontoparietal connectivity, parieto‐occipital α‐oscillation, and attentional capacity.

**Conclusions:**

Patients with aMCI experience significant deterioration in VWM function, particularly during the encoding phase. This deterioration manifests in reduced accuracy and capacity of memory performance, accompanied by a significant decrease in N2pc amplitude, alpha oscillations, and theta‐band connectivity in frontoparietal and fronto‐occipital brain regions. rTMS proves to be a promising intervention that improves VWM, attention, and executive functions. In particular, it supports attention during target selection by increasing N2pc amplitude during encoding, enhancing alpha oscillations for better suppression of irrelevant information, and increasing synchronization in frontoparietal and occipital functional connectivity, which ultimately improves visual working memory.

## Graphical abstract

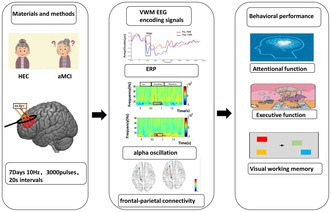

## 1 Introduction

Alzheimer's disease, commonly referred to as senile dementia, is a widespread, age‐related and debilitating disease that affects the elderly population. Epidemiological studies have shown that the prevalence of Alzheimer's disease in people aged 65–74 years is 5% and increases by 5% every 5 years. Beyond the age of 75–84, the prevalence rises to 13.1% and reaches 33.3% in those aged 85 and over [1]. Alzheimer's disease has a profound impact on the quality of life of older people and places a significant burden on families and society. Unfortunately, there are currently no drugs that can effectively reverse or delay the onset and progression of dementia [2]. Patients with aMCI are considered a high‐risk group for clinical conversion to Alzheimer's disease in the elderly population. The annual conversion rate to Alzheimer's disease in people with aMCI is reported to be 10 times higher than in cognitively normal older people [3]. Although the stage of mild cognitive impairment (MCI) is a crucial period for preventing the onset of dementia, there is currently no intervention treatment [4]. In the MCI stage, it is crucial to identify the basic cognitive neural mechanisms underlying the development of cognitive impairment, identify potential intervention targets, and mitigate or delay the onset of dementia.

Research has shown that impaired working memory is a decisive factor in the occurrence of memory disorders [5]. It has been proven that visual working memory plays a fundamental role in various advanced cognitive processes, for example, logical thinking and problem solving [6]. It is a relay station that connects the brain to the outside world and is closely linked to the performance of everyday activities. VWM impairment has been identified as a reliable predictor of progression from MCI to Alzheimer's disease [7]. It is of great scientific importance to investigate the mechanism of neuronal circuits for memory impairment in aMCI from the perspective of VWM. It is of great scientific importance to investigate the mechanism of neural circuits of memory impairment in aMCI from the perspective of VWM. Previous studies have shown a significant positive correlation between VWM and general cognitive function [8]. On this basis, we hypothesize that impaired visual working memory may be a central factor in the early memory deficits observed in patients with aMCI. Furthermore, improving visual working memory in patients with aMCI could be a crucial starting point for improving the overall cognitive function of these patients.

rTMS is an advanced and non‐invasive neural stimulation technique that uses rapidly alternating magnetic fields to stimulate the cerebral cortex and modulate brain activity. It is able to modulate neuronal function in real time during the stimulation period and also exerts a remarkable offline modulation effect that lasts even after stimulation has ceased [9]. The DLPFC is usually selected as the focal region for rTMS in therapeutic interventions for the treatment of MCI or Alzheimer's disease. This specific brain region is widely recognized for its significant involvement in the first cognitive functions affected by Alzheimer's disease, such as attention, executive function and working memory [10]. Research has shown that the DLPFC is involved in the encoding and manipulation of visual working memory and plays a crucial role in the retention of information by retrieving stored data, suppressing irrelevant stimuli, updating information, and exerting top‐down control over the memory process [11, 12, 13]. Several studies have shown that high‐frequency repetitive transcranial magnetic stimulation (HF‐rTMS) targeting the left DLPFC can improve cognitive abilities, including working memory, attention, and executive control, in both healthy individuals and those with neuropsychiatric disorders [14, 15]. Nevertheless, previous studies that have looked at improving working memory through transcranial magnetic stimulation (TMS) have primarily investigated younger people, and there remains a lack of comprehensive research into the neural mechanisms underlying working memory in older people, particularly those with cognitive impairment. Further research is needed to understand how rTMS enhances visual working memory by activating the DLPFC, including the specific phase of information processing in visual working memory and the corresponding changes in cortical electrical activity.

Electroencephalography (EEG) provides valuable insights into the dynamics of working memory processes in the human brain in the millisecond range and offers better time and frequency resolution compared to other neuroimaging methods such as functional magnetic resonance imaging (fMRI). By capturing VWM‐related features of brain activity, EEG enables the reconstruction of oscillatory neuronal activity and functional brain networks. This enables a more comprehensive understanding of the mechanisms underlying working memory [16]. The investigation of event‐related potentials (ERPs) in different brain regions during the VWM task provides a crucial basis for analyzing the cognitive neural mechanisms involved. Studies have shown that information processing in the brain sequentially activates different regions in the frontal, parietal, and occipital lobes, with corresponding ERPs observed. One of the electrophysiological components investigated is the early P1, which is characterized by a typical peak between 80 and 130 ms. This component is closely related to attentional control and mainly affects the dorsolateral prefrontal cortex [17]. The N2pc component then manifests itself around 200–300 ms after the onset of the stimulus. It is postulated that it signifies the spatially selective processing of the ongoing task‐related stimulus [18] and exhibits primary localization in the occipital cortex [19]. The CDA component (contralateral delay activity) begins to emerge around 300 ms and persists until the recognition object is presented. It serves as a neural index reflecting the target or outcome of the control process. The CDA, which is mainly localized in the occipital cortex, is the only currently recognized neural metric for monitoring the object information stored in the VWM in real time and for accurately assessing the amount of objects stored in the VWM [20]. Nevertheless, previous studies have focused predominantly on young people, leaving a gap in understanding the changes in ERP component properties during the different phases of VWM processing in patients with aMCI. Also, the effects of rTMS on the modulation of ERP component amplitude values during the different phases of VWM processing remain to be clarified. Further studies are needed to close these research gaps.

EEG oscillations are a valuable tool for investigating the underlying neural mechanisms that underlie a variety of cognitive processes. The processing of VWM depends on the intricate interplay of neuronal oscillations in different frequency bands. In particular, theta oscillations have been shown to be a crucial player in VWM, orchestrating the encoding of different types of information [21]. Furthermore, these theta oscillations are widely regarded as crucial for the effective functioning of memory [22, 23]. The amplification of alpha oscillations could be related to the cortical inhibition mechanism [24], that could ensure the internal maintenance of information by inhibiting the processing of potentially disruptive information [25]. Activity within the beta and gamma frequency bands is thought to play an important role in the encoding, retrieval, and storage of stimulus material [26]. The unexplored role of oscillations in certain frequency bands in cognitive neuronal mechanisms, especially in aMCI, prompts further investigation. There are still uncertainties regarding top‐down adaptations, neuronal oscillations, and the functional connectivity of the brain in different frequency bands during different processing phases.

In this study of aMCI patients and healthy elderly controls, a conventional change detection paradigm and a real‐time ERP task were used to assess and investigate VWM function. A comprehensive analysis of performance oscillations and functional connectivity of the brain in the context of VWM tasks aims to elucidate the correlation between visual working memory impairment in aMCI patients and ERP components, performance oscillations, and functional connectivity. This methodologically rigorous approach aims to improve our understanding of the cognitive neural mechanisms underlying visual working memory impairment in aMCI.

## 2 Materials and Methods

### 2.1 Participants

The participants in this study were selected from the outpatient clinic of the neurology department of Tongji Hospital. These individuals complained of memory loss. A total of 40 participants met both the inclusion and exclusion criteria and were included in the study. The study included 25 patients diagnosed with aMCI (13 males and 12 females, with a mean age of 70.67 ± 7.70 years) and 15 subjects in the healthy elderly control (HEC) group (7 males and 8 females, with a mean age of 68.36 ± 8.60 years). The ethics committee of Tongji Hospital in Shanghai granted ethical approval for this study, and all participants gave their informed consent.

#### 2.1.1 Inclusion Criteria for aMCI Patients

1. Age range from 50 to 85 years.
2. Voluntary signing of the informed consent form.
3. Neuropsychological assessment meeting the criteria of the 2011 NIA‐AA [27] diagnostic standards, including complaints of gradual cognitive decline or informed reports of cognitive decline. MMSE scores: illiterate ≤ 17 points, elementary school students ≤ 20 points, high school students and above ≤ 24 points; or MoCA‐B: elementary school students and below (6) ≤ 19 points, middle school students (12) ≤ 22 points, academics ≤ 24 points. CDR = 0.5, no fulfillment of the criteria for dementia; impairment of the cognitive domain that fulfills the following criteria: a. Presence of impairment of the cognitive domain memory; b. Impairment of more than one indicator in cognitive domains other than memory (> 1SD).

#### 2.1.2 Inclusion Criteria for HEC

1. Age 50–85 years old, gender unlimited;
2. Willingness to sign the informed consent form.
3. Compliance with safety standards for TMS treatment.
4. Normal objective memory assessment, normal overall cognitive test: MMSE: illiterate > 17 points, elementary school students > 20 points, high school students and above > 24 points; MoCA‐B: elementary school students and below > 19 points, high school students > 22 points, university students > 24 points; CDR: 0;
5. All tests of cognitive domains are in the normal range or only one test is showing deterioration in score.
6. Maintenance of general cognitive function and daily living skills.

#### 2.1.3 Exclusion Criteria

1. Patients who have been clinically diagnosed with dementia and have a history of cerebrovascular disease.
2. Impaired consciousness, severe visual, hearing, or speech disorders, or other physical illnesses that severely impair neuropsychological tests.
3. Severe underlying diseases of the liver, kidneys, hematopoietic system, endocrine system, mental illness, epilepsy, Parkinson's disease, diabetes, excessive alcohol consumption, drug abuse, or malignant tumors.
4. Patients with contraindications to TMS, including a history of metal implants such as metal clips, plates, or rods; brain surgical implants such as electrodes, pacemakers, or drug pumps; hearing implants; bullets; or metal fragments.

### 2.2 Evaluation of the Baseline Data

Prior to enrollment in the study, all participants signed an informed consent form and underwent relevant tests, including CT or MRI scans of the head and biochemical blood tests for folic acid, vitamin B12, thyroid function (FT3, FT4, TSH), syphilis, and HIV antibodies. Participants also completed a battery of neuropsychological tests, including MMSE, MoCA‐B, CDR, memory assessment (HVLT, Merriam‐Webster Logical Memory), performance function assessment (STT, number symbol transformation, Stroop), language assessment (Boston word naming and word fluency), visuospatial function assessment (Rey‐O complex graphics), and instrumental activities of daily living (IADL). The Hamilton Anxiety Rating Scale (HAMA) and the Hamilton Depression Rating Scale (HAMD) were used to assess anxiety and depression symptoms and their severity. The investigators knew nothing about the participants' treatment.

### 2.3 Visual Working Memory Task

**FIGURE 1:**
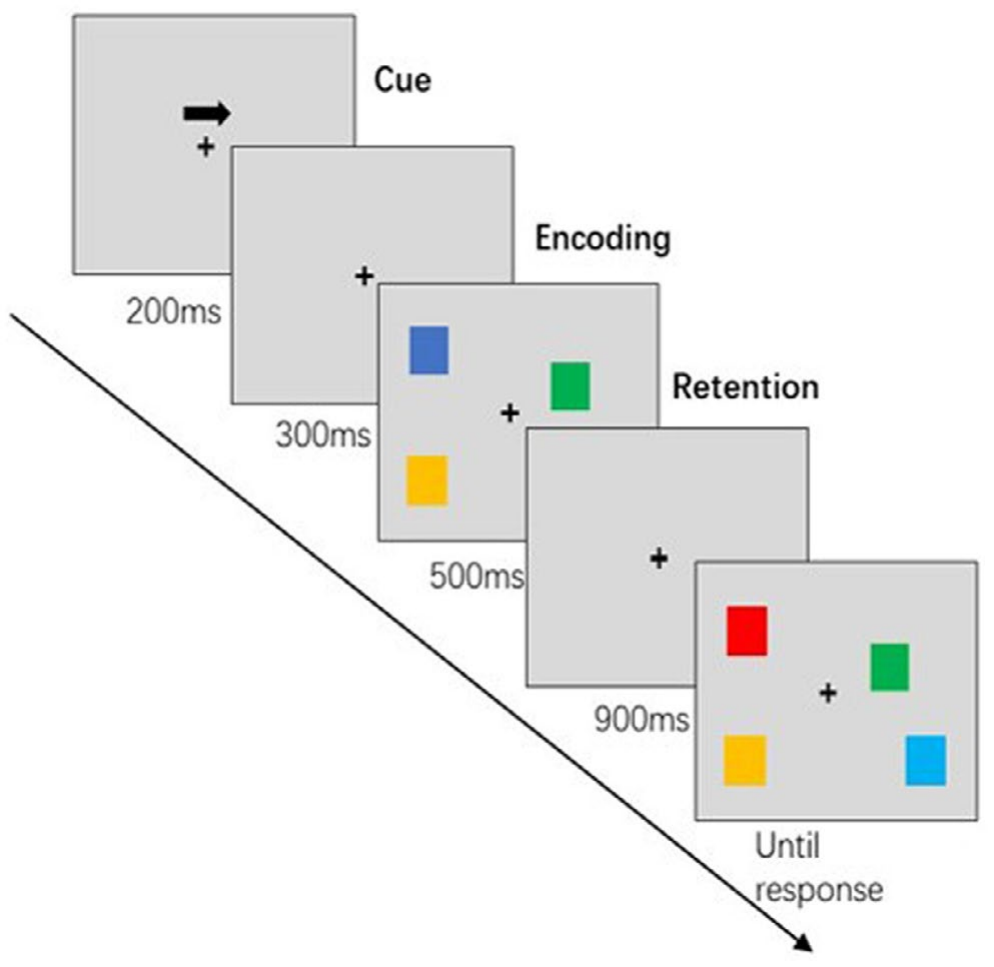
VWM Task Flowchart. Example of a visual memory test for the right hemifield, indicating the duration of the cue (200 ms) with fixation (300 ms), the duration of memory encoding (500 ms), the duration of storage (900 ms) and the duration of retrieval (until response).

The subjects were seated in a quiet room about 60 cm from a computer screen. The VWM task was performed with the Eprime software (3.0). In this study, an extended task was used to detect changes in visual working memory [28] (see Figure 1 for the flowchart of a single VWM test). At the beginning, a left or right arrow appeared on the gray screen indicating the point of gaze. Participants were instructed to focus their eyes on the focal point while paying attention to the direction of the arrow in their peripheral vision. A pattern sequence with an equal number of small squares in different colors to the left and right of the fixation point was then presented for 500 ms. During this period, participants had to focus on the central fixation point and recall the color of the small squares on the side indicated by the arrow from their peripheral memory. The example sequence was then faded out, and the recognition sequence appeared after a presentation of the fixation point for 900 ms. Blocks of color appeared on either side of the fixation point, indicating the position and quantity of the example sequence. Participants were asked to determine whether the color of the small square on the side to which the arrow pointed had changed compared to the sample sequence. The stimulus sequence consisted of 50% variable and 50% constant conditions, with the key assignment being the same for all participants. The recognition sequence disappeared as soon as the participant made a judgment so that the next trial could begin. The interval between trials was set at 2000 ms. Each block consisted of 50 trials, totaling 100 trials, with a break every 50 trials. Prior to the formal experiment, participants completed practice sessions to ensure that they fully understood the task before the actual study began.

### 2.4 TMS Stimulation Protocol

rTMS was performed with an active figure‐8 coil (BY90A, Shenzhen Yingchi Technology Co. Ltd., Shenzhen, China) and pulsed magnetic stimulation (M‐100 Ultimate, Shenzhen Yingchi Technology Co. Ltd., Shenzhen, China). Resting Motor Threshold (RMT) refers to the minimum single‐pulse intensity required by the Motor Evoked Potential (MEP) to induce a peak movement of 50 μV and above when the patient is at rest. The MEP indicates the contraction potential recorded at the corresponding target tendon after rTMS stimulation of the motor cortex. During the examination, the subject sits upright and relaxed on a chair and places their hands flat on their legs. The center of the coil is placed tangentially over the subject's left motor cortex and triggers a single‐pulse stimulation to observe the movement of the right hand. The threshold was gradually lowered when involuntary movements occurred until the maximum threshold was reached at which the involuntary movements ceased, representing the RMT. The parameters of TMS stimulation included targeting the left DLPFC (F3 electrode). The experimental group received stimulation at a frequency of 10 Hz. Each rTMS session consisted of 30 stimulation sequences, each consisting of 100 stimulation pulses. The interval between two consecutive sequences was 20 s. Each treatment comprised a total of 3000 pulses, with the stimulation intensity set to 100% of the subject's RMT. The subjects underwent a one‐week rTMS treatment. They received high‐frequency rTMS once a day with the stimulation parameters described.

### 2.5 EEG Recording and Signal Pre‐Processing

EEG data for the VWM task was recorded immediately before the initiation of the rTMS intervention and again on the day it concluded. This precise timing was deliberately chosen to capture the immediate neural effects induced by the treatment, enabling us to assess both baseline neural activity and the dynamic changes occurring as a result of the intervention. By collecting data at these two critical time points, the study provides a comprehensive understanding of how rTMS modulates brain function and its potential therapeutic impact on targeted neural pathways. EEG recordings were performed in a quiet room using a NeuroScan SynAmps2 amplifier (SynAmps2TM Mode 8050, NeuroScan) equipped with 64 individual Ag/AgCl scalp electrodes. Four facial electrodes were strategically placed around each eye to measure eye movements. The electrodes were grounded to GND and referenced online to the electrodes between CZ and CPZ. Before recording, the impedance was kept below 10 kΩ. The sampling rate was set to 1000 Hz, and the signals were recorded with an online bandpass filter in the range of 0.01 Hz to 250 Hz. Offline preprocessing of the raw EEG data was performed using MATLAB software (version R2013a) and the EEGLAB toolbox (version 14.1.1 [29]). The Preprocessing steps included: (1) removal of redundant channels, such as HEO, VEO, M1, M2, CB1, CB2, and trigger; (2) band‐pass filtering in the range of 0.1 Hz to 95 Hz, followed by the use of notch filters to eliminate 50 Hz (filter range 49 Hz to 51 Hz) and 100 Hz (the first harmonic of 50 Hz, filter range 99 Hz to 101 Hz) line noise; (3) interpolation of bad channels using spherical or average interpolation to correct excessive noise or drift; For the raw EEG data of Pre_rTMS, 30 out of 40 participants required such interpolation (5 channels for 1 participant, 3 channels for 9 participants, 2 channels for 16 participants, and one channel for 4 participants). More details about the preprocessing of EEG can be found in the Supporting Information. (4) segmenting the data into time segments from 1000 ms before stimulus sequence presentation to 2000 ms after; (5) removing bad segments by visual inspection; (6) A major advantage of Independent Component Analysis (ICA) is its ability to effectively identify specific artifacts, such as eye movement and electrocardiographic artifacts, using topographical maps, trial‐by‐trial distributions, and frequency distributions. These artifacts often exhibit distinct characteristics. Generally, artifact‐related components are concentrated in higher frequency bands (e.g., above 30 Hz), while neural signals are predominantly found in lower frequency bands (e.g., below 20 Hz). By integrating information from topographical maps, power distribution plots, and component waveforms, these artifacts can generally be identified, with 1–2 components usually associated with blinks. As for muscle artifacts, on the other hand, they are more frequently observed in the temporal or prefrontal regions. In energy distribution plots, they often present two peaks within the 20–50 Hz range, while their component waveforms show relatively regular spiky patterns. The number of muscle artifact components typically ranges from 5 to 10. Other artifacts, such as those caused by head movement, cardiac activity, or sweating, are relatively rare. In summary, approximately 10 artifact components are typically removed from our data, identified through a comprehensive analysis of topographical maps, energy distribution plots, and component waveforms. Applying independent component analysis (ICA) [30] to eliminate independent components containing artifacts, such as eye movements, ECG, etc., and (6) removal of bad segments by visual inspection. (7) removal of extreme values, deletion of study segments with an amplitude greater than ±100 μV; (8) re‐referencing using the average bilateral mastoid M1M2 reference. The quality control criterion for the EEG data was set at a minimum of 50% usable trials (i.e., each EEG data length contained more than 50 trials) for inclusion in subsequent analyses.

### 2.6 Methods of EEG Analysis

#### 2.6.1 Event‐Related Potential Analysis

When analyzing the event‐related potentials (ERPs), the average amplitude at the electrodes in the parieto‐occipital region (PO7/PO8) is calculated by subtracting the average activity of the contralateral side. Both the contralateral and ipsilateral amplitude values are determined by superimposing the activity of the electrodes on the target side. The ERP difference wave is derived by subtracting the ERP waveform of the symmetrically opposite electrode from that of the corresponding electrode in the target region. The opposite waveform of the target is the mean of the electrodes in the left hemisphere when the target is presented in the right visual field and vice versa for the right hemisphere. Similarly, the ipsilateral waveform of the target represents the average activity of the electrodes in the left hemisphere when the target is presented in the left visual field and in the right hemisphere when it is presented in the right visual field. The extracted components include the P1 component during the cue period, the N2pc component during the encoding period, and the CDA component during the maintenance period of visual working memory processing. The mean amplitude values of the contralateral difference wave in the specified time windows (P1: 80 ms‐120 ms, N2pc: 164 ms‐258 ms, CDA: 400 ms‐1000 ms) are then extracted for the subsequent statistical tests.

#### 2.6.2 Event‐Related Spectral Perturbation Analysis

The calculated event‐correlated spectral perturbation is analyzed by wavelet transform using the Morlet wavelet transform [31]. The decomposition is performed in the frequency range of 4–30 Hz in 1 Hz intervals, with a fixed wavelet period of 3 cycles. To facilitate the comparison of spectral energy differences under different conditions, the original energy values obtained after the time‐frequency transformation are subtracted contralaterally from the ipsilateral side. Specifically, this means that the activity of the electrode on the opposite side of the cue (arrow) is subtracted from the corresponding electrode on the same side. A baseline correction is performed by selecting the period 600–400 ms before the stimulus occurs and using the subtraction for the baseline correction. The resulting Event‐Related Spectral Perturbation (ERSP) values (μV^2^/Hz) are determined based on this common baseline. The ERSP values are extracted from frontal (FP1/FP2) and parieto‐occipital (PO7/PO8) regions in different frequency bands—θ (4‐7 Hz), α (8‐13 Hz), β (14‐30 Hz) and γ (31‐80 Hz)—at different stages of visual working memory processing (cue, encoding, retention). These values are then used to perform statistical tests.

#### 2.6.3 Functional Connectivity Analysis

Functional connectivity is analyzed using the weighted period lag index (wPLI), which is based on the period‐based method. The wPLI depends on the distribution of period angle differences and becomes more synchronous the stronger the functional coupling between two channels is. It mitigates the volume conduction caused by connectivity with zero period shift when the electrodes measure the electrical activity from the same source. The connectivity calculation gives values between 0 and 1 for each pair of electrodes at each frequency and time, where 1 represents full synchronization and 0 represents no synchronization. The formula for the wPLI calculation includes the imaginary part of the cross‐spectral density at time t in the complex plane xy. In this analysis, the average wPLI values for the frontal lobe (F3, F1, F5, FC1, FC3, AF3), the parietal lobe (P1, P2, P3, P4, P5, P6, P7, P8) and the occipital lobe (O1, O2, PO3, PO5, PO7, PO4, PO6, PO8) during the different periods of visual working memory processing (cue, encoding, maintenance) in different frequency bands—θ (4‐7 Hz), α (8‐13 Hz), β (14‐30 Hz), γ (31‐80 Hz). This results in a weighted symmetric connectivity matrix (57 × 57) for each subject in each frequency band, with subsequent FDR correction for multiple comparisons in follow‐up tests for significant main effects or interactions.

${\text{WPLI}}_{\mathrm{xy}}=\frac{n^{-1}\sum_{t=1}^{n}|\text{lmag}\left(s_{\mathrm{xyt}}\right)|\mathrm{sgn}\;\left(\text{imag}\left(s_{\mathrm{xyt}}\right)\right)}{n^{-1}\sum_{t=1}^{n}|\text{imag}\left(S_{\mathrm{xyt}}\right)|}$

### 2.7 Behavioral Analysis

Behavioral data extracted from the VWM files recorded by the Eprime3.0 software include accuracy (correct count), reaction time (from the appearance of the recognition sequence to the subject's key press, excluding trials exceeding 3000 ms; and the mean of the remaining trials); and visual memory capacity, represented by the *K* coefficient. The *K* coefficient [32] is calculated according to the following formula: *K* = memory load × (hit rate − false alarm rate)/(1—false alarm rate). It indicates how many items a person can remember when presented with a certain number of items. These extracted metrics are then statistically tested.

### 2.8 Statistical Analysis

The statistical analysis was carried out using SPSS software (version 20.0, Tongji University, China). All statistical tests are two‐tailed tests, with the significance level set at *p* < 0.05. Descriptive statistics for count data are presented as the number of cases (%), while measurement data are presented as cases, mean, standard deviation, and median. The independent t‐test and chi‐square test were used to assess the differences in demographic characteristics between the two groups. The difference between the two groups was assessed using the independent t‐test. With regard to the therapeutic difference before and after the rTMS intervention, the paired t‐test was used for within‐group comparisons and the independent t‐test for between‐group comparisons at posttest. We performed a correlation analysis between the behavioral performance of visual working memory and the extracted EEG signal features. FDR verification is required for multiple comparisons.

## 3 Results

### 3.1 Demographic Data

Table 1 contains the demographic data of the study participants, including age, gender, years of education, general cognitive assessment (MMSE and MoCA‐), depression assessment (HAMD‐17), and comprehensive neuropsychological tests. The neuropsychological tests cover different domains, such as memory function (immediate recall, delayed recall after 5 min and delayed recall after 20 min in the Hopkins Verbal Learning Test), language function (Boston Naming Test score, verbal fluency score), executive function (STT‐A and STT‐B) and visuospatial function (Rey‐O Complex Figure Test—copying and retrieval). Specific details can be found in Table 1.

**TABLE 1:** Demographic data.

	HEC (*n* = 15)	aMCI (*n* = 25)	*p*
Age	68.36 ± 8.60	69.60 ± 9.33	0.67
Educational years	11.24 ± 4.63	11.93 ± 2.28	0.59
Gender(male/female)	(8/7)	(13/2)	0.82
MMSE	27.20 ± 1.59	23.2 ± 3.48	< 0.001***
MoCA‐B	24.07 ± 3.15	17.96 ± 4.27	< 0.001***
HAMD	4.53 ± 4.37	6.32 ± 5.89	0.16
Hopkins verbal learning test (HVLT) immediate	22.00 ± 4.47	15.16 ± 4.61	< 0.001***
HVLT‐Delayed recall (5 min)	7.80 ± 1.57	3.16 ± 3.34	< 0.001***
HVLT‐Delayed recall (20 min)	7.53 ± 1.69	3.36 ± 3.24	0.001**
Logical memory test ((Wechsler memory scale))	9.80 ± 2.21	6.64 ± 2.83	0.001**
			0.001**
Boston naming test	24.00 ± 2.62	20.98 ± 4.67	0.005**
Verbal fluency test‐vegetables	14.40 ± 2.87	11.00 ± 3.75	0.014*
Shape trails test (STT‐A)	54.47 ± 9.67	82.24 ± 8.17	0.014*
Shape trails test (STT‐B)	134.27 ± 46.14	182.64 ± 70.95	0.094
Rey‐Osterrieth Complex	34.67 ± 1.88	32.76 ± 4.03	0.094
Figure Test (The copy scores)			
Rey‐Osterrieth Complex	20.33 ± 7.56	8.24 ± 7.78	< 0.001***
Figure Test (The recall scores)			

*Note:* The chi‐square test was used for the gender composition ratio, and the independent‐samples *T*‐test and the two‐tailed test were used for the values of the other age groups, years of education, and cognitive domains.

* *p* < 0.05.

** *p* < 0.01.

*** *p* < 0.001.

### 3.2 Behavioral Results

**FIGURE 2:**
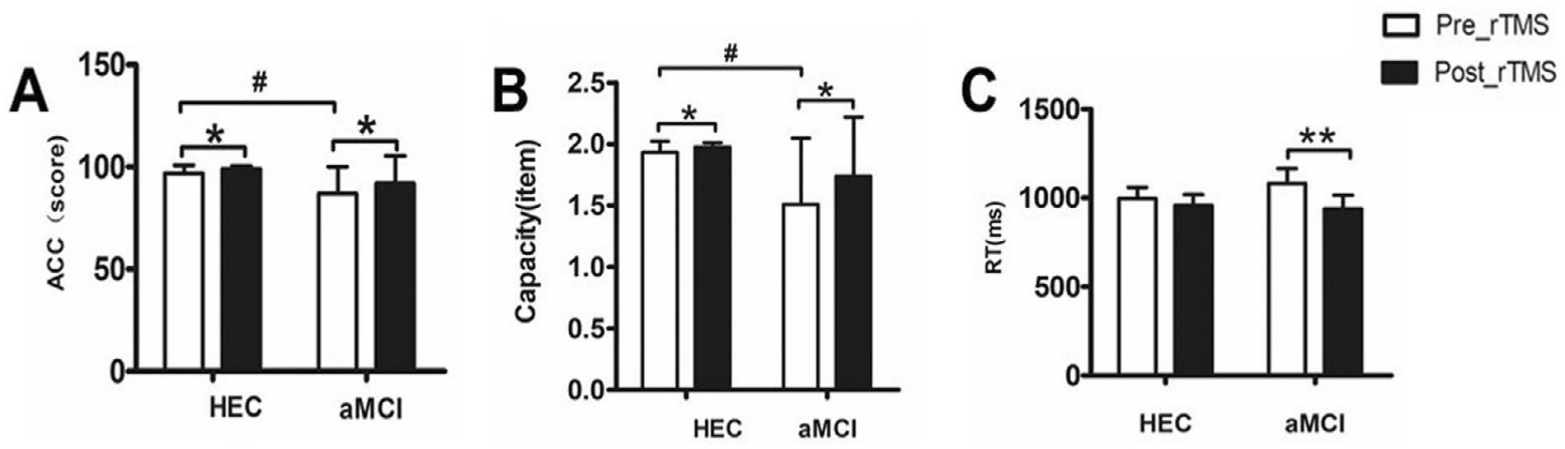
Comparison of VWM performance differences between the HEC group and the aMCI group before and after the rTMS intervention. Pre_rTMS: Before the rTMS intervention; Post_rTMS: After the rTMS intervention; (A) shows the differences in the accuracy of the VWM task between the aMCI group and the HEC group before and after the rTMS intervention. (B) Shows the differences in the capacity of the VWM task between the aMCI group and the HEC group before and after the rTMS intervention. (C) Shows the differences in reaction time in the VWM task between the aMCI group and the HEC group before and after the rTMS intervention. The significant differences between the aMCI group and the HEC group before the rTMS intervention are labeled with #. The significant differences between the aMCI group and the HEC group before and after the rTMS intervention are marked with * (the symbol *p < 0.05, while **p < 0.01).

As shown in Figure 2. Before rTMS, patients with aMCI showed significantly lower accuracy and memory capacity in VWM tasks compared to the HEC group (accuracy: 86.96 ± 12.97 vs 96.87 ± 3.94, *p* = 0.007; memory capacity: 1.51 ± 0.54 vs 1.93 ± 0.094, *p* = 0.005). However, significant improvements were observed in the aMCI patients after 7 days of rTMS intervention. VWM task accuracy increased (86.96 ± 12.97 vs. 91.96 ± 13.35, *p* = 0.017), memory capacity improved (1.51 ± 0.54 vs 1.81 ± 0.32, *p* = 0.013) and reaction time decreased (939.628 ± 382.45 vs. 1081.37 vs 422.64, *p* = 0.007).

**FIGURE 3:**
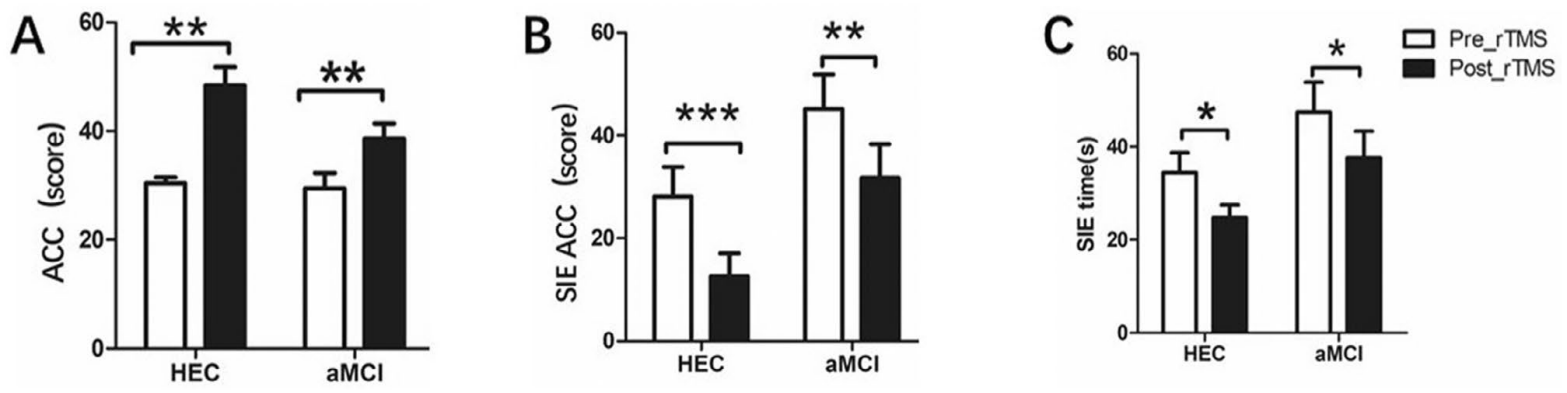
Comparison of attention and executive function differences between the HEC group and the aMCI group before and after the rTMS intervention. (A) Shows the differences in accuracy between the aMCI group and the HEC group before and after the rTMS intervention. The graph shows the accuracy rates achieved by both groups on the digit symbol substitution test and highlights any significant changes resulting from the rTMS intervention. Figure (B) shows the differences in Stroop interference effects(SIE)accuracy(ACC) (The difference between the number of correct responses in Stroop_C and Stroop_B.) between the aMCI group and the HEC group before and after the rTMS intervention. The graph shows the accuracy rates achieved in the Stroop test for both groups and emphasizes all significant changes resulting from the rTMS intervention. (C) Shows the differences in Stroop interference effects(SIE)time (the difference between the time required in Stroop_C and Stroop_B.) between the aMCI group and the HEC group before and after the rTMS intervention. The diagram shows the accuracy rates achieved in the Stroop test for both groups and emphasizes all significant changes resulting from the rTMS intervention. The significant differences before and after the rTMS intervention are marked with * (the symbol *p < 0.05, while **p < 0.01).

As shown in Figure 3, in the Stroop_C task with interference effects, we observed that rTMS significantly reduced the total time to complete the task in both the aMCI patients and the HEC groups (aMCI group: 93.12 ± 33.14 vs 80.52 ± 32.29, *p* = 0.001; HEC group: 76.67 ± 21.02 vs 62.47 ± 17.54, *p* = 0.001). In addition, rTMS significantly reduced the SIE time (aMCI group: 45.12 ± 33.60 vs 31.76 ± 28.78, *p* = 0.002; HEC group: 28.13 ± 22.15 vs 12.53 ± 17.59, *p* < 0.001) and improved the number of correct responses (aMCI group: 47.44 ± 32.27 vs 37.60 ± 28.78, *p* = 0.033; HEC group: 34.40 ± 16.73 vs 24.73 ± 10.82, *p* = 0.023).

### 3.3 Effects of rTMS on ERP Components

**FIGURE 4:**
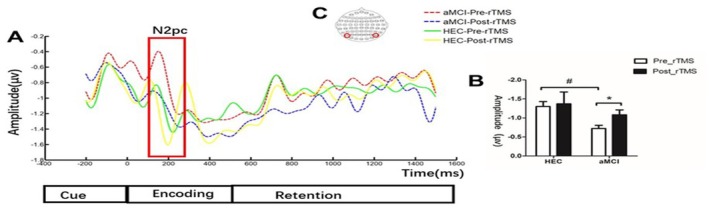
Comparison of N2pc amplitude differences between the HEC group and the aMCI group before and after the rTMS intervention. (A) Illustrates the changes in ERP waveforms between the HEC group and the aMCI group before and after the rTMS intervention. The red dotted box represents the extraction of the N2pc time window region (164–258 ms). The blue line represents the ERP waveform before rTMS in the aMCI group, while the red line represents the ERP waveform after rTMS in the aMCI group. Similarly, the green line represents the ERP waveform before rTMS in the HEC group, while the yellow line represents the ERP waveform after rTMS in the HEC group. (B) Shows a bar chart comparing the differences in N2pc amplitude between the aMCI group and the HEC group both before and after the rTMS intervention. The graph illustrates the magnitude of the N2pc component in both groups and highlights any significant differences between them. (C) Shows the selection of electrodes from specific brain regions of interest (ROIs) for the ERP analysis. The significant differences between the aMCI group and the HEC group before the rTMS intervention are marked by #. The significant differences before and after the rTMS intervention are marked by *.

We performed an ERP analysis of the different periods of VWM processing and found significant differences only during the encoding period. Figure 4 shows that the amplitude of N2pc was significantly lower in patients with aMCI compared to the HEC group (−0.72 ± 0.41 vs. −1.30 ± 0.51, *p* < 0.001). However, after 7 consecutive days of rTMS intervention, we observed a significant increase in the amplitude of N2pc in patients with aMCI (−0.72 ± 0.41 vs. 1.08 ± 0.62, *p* = 0.028), while no significant difference was observed in the HEC group (−1.30 ± 0.51 vs. 1.36 ± 1.19, *p* = 0.85).

### 3.4 Effects of rTMS on Time‐Frequency Distribution

**FIGURE 5:**
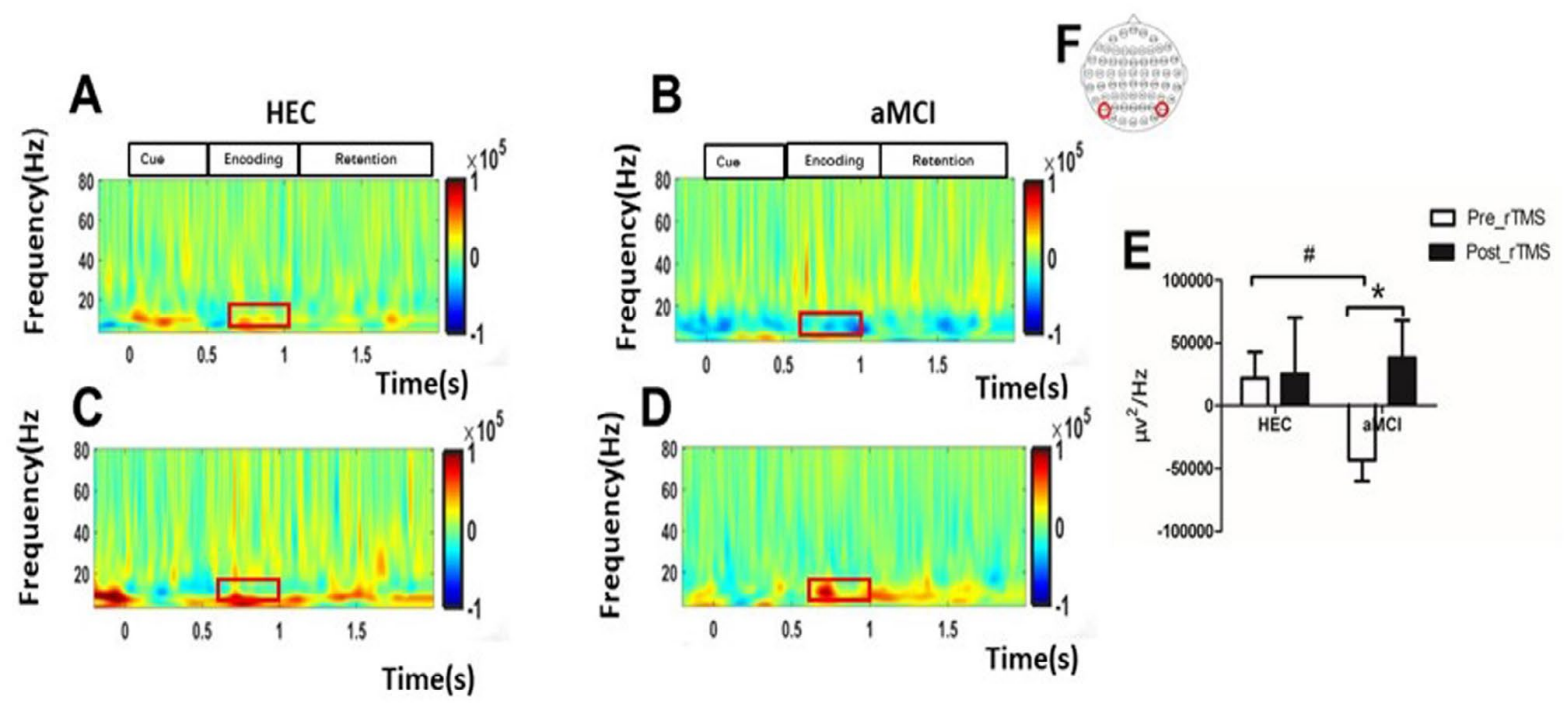
Comparison of alpha oscillation differences between the HEC group and the aMCI group before and after the rTMS intervention. (A) shows the time‐frequency distribution of the parietal and occipital regions in the HEC groups before repetitive transcranial magnetic stimulation (rTMS). (B) Shows the time‐frequency distribution of the parietal and occipital regions in the aMCI groups before rTMS. (C) shows the time‐frequency distributions of the parietal and occipital regions in the HEC groups after rTMS. (D) shows the time‐frequency distributions of the parietal and occipital regions in the aMCI groups after rTMS. Red dashed box: The mean ERSP of the alpha band (8–12 Hz) selected during the encoding period (0.68–1 s), and the upper right corner shows the flag plot of the selected electrode (PO7/PO8). (E) illustrates the differences in alpha oscillations during the encoding phase of visual working memory (VWM) between the groups and before and after rTMS. (F) shows the selection of electrodes from specific brain regions of interest (ROIs) for the time‐frequency analysis. The significant differences between the aMCI group and the HEC group before the rTMS intervention are marked by #. The significant differences before and after the rTMS intervention are marked by *.

Before rTMS intervention, we observed that parieto‐occipital alpha oscillations during the VWM encoding period were significantly lower in patients with aMCI compared to the HEC group (−0.32 × 105 ± 0.82 × 105 vs. 0.21 × 105 ± 0.81 × 105, *p* = 0.049). However, after 7 consecutive days of rTMS intervention, there was a significant enhancement of parieto‐occipital alpha oscillations during the VWM encoding period in patients with aMCI(−0.32 × 105 ± 0.38 × 105 vs. −0.48 × 105 ± 0.48 × 105, *p* = 0.047). As shown in Figure 5.

### 3.5 Effects of rTMS on Functional Connectivity

**FIGURE 6:**
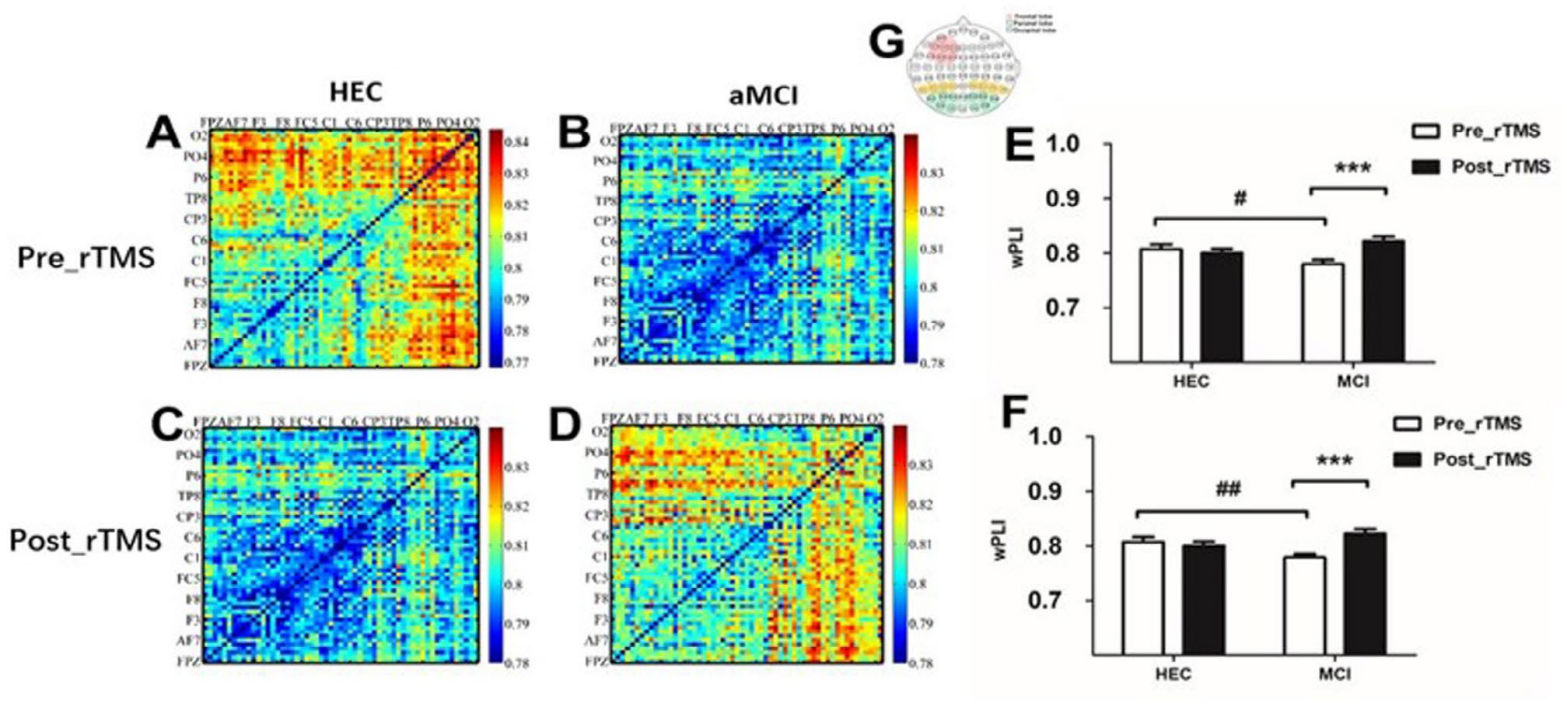
Comparison of Functional Connectivity differences between the HEC group and the aMCI group before and after the rTMS intervention. (A) shows matrices depicting the functional connectivity patterns during the encoding of visual working memory in HEC prior to rTMS. (B) shows the matrices of functional connectivity during VWM encoding in the aMCI groups prior to rTMS. (C) shows the matrices of functional connectivity during VWM encoding in the HEC groups after rTMS, while (D) shows the matrices of functional connectivity during VWM encoding in the aMCI groups after rTMS. To further analyze the differences between the groups, (E) shows a bar chart comparing the variations in frontal–parietal connectivity. (F) also shows a bar chart comparing the differences in frontal‐occipital connectivity between the groups. (G) illustrates the selection of electrodes from specific brain regions of interest (ROIs) for the wPLI analysis. The frontal lobe electrodes are shown in pink, while the parietal lobe electrodes are represented by the yellow area. The synchronization index for this particular brain region was calculated by determining the values of the wPLI from multiple recordings. The significant differences between the aMCI group and the HEC group before the rTMS intervention are indicated by # (the symbol ^#^ *p* < 0.05, while ^##^ *p* < 0.01). The significant differences before and after the rTMS intervention are marked by * (the symbol ***p < 0.001).

Before the rTMS intervention, we observed that patients with aMCI had significantly lower fronto‐parietal connectivity in the θ‐band compared to the HEC group during the VWM encoding period (fronto‐parietal connectivity: 0.779 ± 0.04 vs 0.807 ± 0.03, *p* = 0.038; fronto‐occipital connectivity: 0.778 ± 0.033 vs 0.81 ± 0.027, *p* = 0.002). However, after 7 consecutive days of rTMS intervention, patients with aMCI showed significant improvement in fronto‐parietal connectivity and fronto‐occipital connectivity in the θ‐band during the VVWM encoding period (fronto‐parietal connectivity: 0.779 ± 0.04 vs 0.821 ± 0.046, *p* < 0.001; fronto‐occipital connectivity: 0.778 ± 0.033 vs 0.822 ± 0.046, *p* < 0.001). As shown in Figure 6.

### 3.6 Correlation Analysis

The decrease in visual working memory capacity correlated significantly with a decrease in θ‐band frontoparietal connectivity during the encoding period (*r* = 0.472, *p* = 0.002). The alpha oscillation in the parieto‐occipital region showed a significant correlation with attentional ability (*r* = −0.432, *p* = 0.005). We compared the changes in behavioral and electrophysiological indicators before and after the rTMS intervention (The ratio of measurements after rTMS to those before rTMS) and found that the magnitude of improvement in visual working memory capacity was closely associated with the magnitude of improvement in the θ‐band of the frontoparietal junction during the encoding period (*r* = 0.329, *p* = 0.038). In addition, the improvement in alpha oscillation in the parieto‐occipital region was significantly correlated with the improvement in attentional performance (*r* = 0.325, *p* = 0.041).

## 4 Discussion

### 4.1 Apparent Impairment of Visual Working Memory in aMCI Patients

Visual working memory refers to the temporary storage and processing of visual information by the brain for subsequent cognitive processing [33]. It serves as the basis for various complex cognitive processes. Our study showed that patients with aMCI performed VWM tasks with significantly lower accuracy and memory capacity than healthy older controls. Studies have shown that differences in visual working memory capacity are partly due to differences in the attentional processes involved in selecting relevant information and filtering out irrelevant information [34]. Irrelevant information consumes unnecessary capacity, and individuals with low memory capacity tend to encode irrelevant information more than individuals with high memory capacity [8]. Therefore, the efficiency of filtering out irrelevant information could be a crucial factor in limiting the capacity of working memory [35]. Earlier models of working memory assume that differences in working memory performance are primarily due to changes in executive control mechanisms [36]. Studies have shown that subjects with low working memory capacity show significant deficits in the suppression of irrelevant information [37], and there is a close relationship between working memory and attentional control [38]. Older people are more likely to be distracted by distracting information [39]. Our study also found a remarkable positive correlation between reduced visual working memory capacity and impaired executive functions. Consequently, we hypothesize that the decrease in visual working memory observed in aMCI patients may be associated with a significant reduction in their ability to suppress irrelevant information, as well as with a weakening of attention and executive control. However, behavioral performance alone can only provide a general assessment of task performance and cannot provide insight into the underlying neural mechanisms. Therefore, investigating the characteristics of visual working memory impairment in aMCI patients at different stages from an electrophysiological perspective is of great scientific importance and valuable for clinical practice to understand the underlying mechanisms of cognitive decline.

### 4.2 The Amplitude of N2pc Decreased Significantly in the Encoding Period of VWM Processing in aMCI Patients

The ERP study found that aMCI patients showed a significant reduction in the amplitude of the N2pc component during the encoding phase of visual working memory processing compared to healthy older controls. The N2pc component is characterized by a negative waveform that is enhanced contralaterally on the scalp and typically occurs around 200–300 milliseconds after the onset of stimulation [40]. Extensive research has provided convincing evidence that the N2pc component is not only a reliable indicator of the orientation of visual attention but also shows a strong correlation with the encoding process [41]. Furthermore, studies have consistently shown that the amplitude of the N2pc decreases significantly in the elderly during working memory encoding, suggesting that it may serve as a predictive measure of visual working memory ability in this particular population [42]. The N2pc component proves to be a valuable tool for clarifying the involvement of attentional processes in the encoding of VWM [43]. It has been shown that impaired attention leads to reduced encoding of the VWM and consequently to a reduced capacity of the VWM [42]. The N2pc component associated with selective attention specifically reflects the complicated process of target selection. It has been suggested that the amplitude of N2pc may indicate individual differences in the capacity of visual working memory [44, 45]. Our results suggest that patients with aMCI exhibit impaired selective attention and lower utilization of visual working memory resources, resulting in a reduced ability to select targets [46]. Department of Neurology, Shanghai Changhai Hospital, Second Military Medical University, Shanghai, People's Republic of China [47].

We hypothesize that the impairment of visual working memory in amnestic mild cognitive impairment (aMCI) is due to reduced allocation of attentional resources, impaired selective attentional control, reduced target retrieval efficiency, and weakened encoding capacity. Together, these elements contribute to the observed decline in visual working memory capacity.

### 4.3 VWM Task‐Related α‐Band ERD Enhancement in Patients With aMCI

During the memory encoding phase of VWM processing, an increase in ERD can be observed in the alpha band in the parieto‐occipital region. Previous research has shown that alpha oscillations play a role in the suppression of irrelevant information, and some studies have suggested that the suppression of alpha oscillations reflects cortical excitability [48]. The decrease in alpha oscillations during the encoding process indicates that visual processing is in an active state [49]; this suggests that people with aMCI may need to make more cognitive effort to compensate for the decline in their cognitive function [50, 51]. The study found that reduced alpha oscillatory power correlated with a significant decrease in the ability of Alzheimer's patients to suppress distracting information. It is hypothesized that this increased susceptibility to interference is responsible for the reduced cognitive control and memory impairment observed in older adults without cognitive impairment [52]. The EEG of the healthy aging brain also shows a progressive decline in alpha power [53]. Several studies have shown a strong correlation between the event‐related desynchronization (ERD) of alpha oscillations in the frequency range of 8–14 Hz and memory function [54, 55]. The decrease in low‐frequency power serves as an indicator of the active involvement of the cortical modules in the complicated processes of memory encoding and storage. Conversely, desynchronization of alpha‐band oscillations plays a crucial role in improving the ability of the neocortex to provide important feedback information [56]. In addition, our supplementary analysis revealed a strong correlation between the decrease in alpha oscillations and the impairment of executive attention function, further supporting our hypothesis. It is known that alpha oscillations are associated with the allocation of attentional resources. Individuals with aMCI have deficits in attention and visual working memory, and the reduction in alpha‐band activity could disrupt the allocation of attentional resources, negatively impacting performance on visual working memory tasks. The decrease in attention could also be a major factor in the decline of visual working memory [57]. Attention plays a central role in the encoding of information, as it improves the neuronal representations of visual stimuli and the perceptual accuracy of these representations [58]. The accuracy of information encoding is a key predictor of visual working memory impairment [59]. The decline in VWM performance generally observed with increasing age can be attributed to a decrease in the accuracy of encoding or retention processes [60].

The impairment of visual working memory in aMCI results from reduced top‐down regulation of alpha oscillation during encoding, which hinders effective consolidation of information. Impaired attentional capacity and impaired inhibitory control further contribute to the observed deterioration in behavioral performance in people with aMCI.

### 4.4 Synchronization of Frontal–Parietal and Occipital Connections Decreased in the VWM Processing Period of Memory Encoding in aMCI Patients

The frontal and parietal networks are considered important cognitive control networks that play a crucial role in regulating brain activity and general cognitive function. In particular, the frontoparietal network is responsible for the flexible regulation of the activity of other functional networks, which emphasizes its central role in cognitive control processes [61]. A review of 75 fMRI studies showed that patients with mild cognitive impairment have lower activity in the frontal–parietal cortex compared to healthy controls [62]. Frontal connectivity plays a crucial role in the processing of visual working memory. Research has shown that increased synchronization between frontal and parietal regions is associated with the successful storage of a greater amount of information in working memory [53, 63]. The ability of the frontal–parietal network to maintain continuous activity relies on the synchronized and structured activity that occurs within different frequency ranges [64]. Numerous studies have consistently reported a decline in performance on VWM tasks and observed network abnormalities during VWM task processing in patients with aMCI. However, there is currently no consensus on the specific neural mechanism underlying this phenomenon. Previous research suggests that the frontal–parietal network, the visual system network, and the dorsal attention network are activated during visual working memory tasks, particularly during the encoding and recognition phases [65]. From the results of this study, it can be deduced that the impaired task performance of the test subjects could be due to disturbed network oscillations, reduced synchronization, and weakened connectivity. Attentional processes are also crucial in visual working memory tasks, particularly in the input, encoding, and maintenance of visual information related to the task [66]. Behavioral performance results suggest that people with aMCI also have difficulties with attention resource allocation, attention selection, and attention control. These difficulties may impair their functional performance on tasks by disrupting the top‐down loop of attentional regulation, which ultimately affects their VWM behavioral performance.

### 4.5 Brief Summary

Patients with aMCI show a remarkable deterioration in visual working memory, especially during the encoding phase. This deterioration can be attributed to deficits in the allocation of attentional resources, selective attention, and executive control skills. Individuals with aMCI experience a decrease in α‐oscillations in the parieto‐occipital region, resulting in reduced attention and an impaired ability to filter out irrelevant information. In addition, impaired synchronization of the frontal–parietal junction, which plays a crucial role in the processing of VWM, further contributes to the impairment of visual working memory in patients with aMCI.

In the second part of this study, we investigated the effects of high‐frequency (10 Hz) rTMS on cognitive functions, especially visual working memory, in patients with aMCI by targeting the left DLPFC. We also investigated the electrophysiological mechanisms underlying the changes in task‐related EEG signals during visual working memory. Our results showed that rTMS has the potential to improve visual working memory, attention, and executive function. In particular, rTMS showed significant improvements in ERP components (N2pc) associated with the encoding period of visual working memory, as well as alpha oscillations and functional connectivity in the fronto‐parietal and occipital regions.

### 4.6 Effects of rTMS on Visual Working Memory

Our study showed that both aMCI patients and older people with rTMS can significantly improve their accuracy and memory capacity in VWM tasks. In terms of response, rTMS has a more pronounced effect on improving aMCI in the elderly, but the extent of improvement is limited in healthy elderly individuals. This may be due to the marked impairment of executive functions in older people with aMCI and the limited effect of rTMS in healthy older people with relatively intact cognitive functions [67]. These findings suggest that rTMS not only repairs the deficits associated with aMCI but also enhances cognitive performance in healthy elderly individuals. This further underscores the neuroplastic potential of the cerebral cortex [68]. Reaction time, which is usually associated with the speed of information processing [69], serves as an important metric in cognitive evaluations. rTMS can reduce reaction times in elderly individuals with aMCI by stimulating the DLPFC affected by executive function decline. Patients with aMCI are characterized by a decline in processing speed and neural efficiency [70]. rTMS enhances neuroplasticity and promotes neural connectivity, helping to compensate for these deficits and leading to improved reaction times. In contrast, healthy elderly individuals typically have higher cognitive reserve and intact brain function [71]. As a result, their cognitive systems are already operating at an optimal level, making the effects of rTMS less pronounced. The treatment may yield limited benefits due to a “ceiling effect,” as any further improvement is restricted by their already optimal baseline cognitive performance.

Our study provides evidence that rTMS can effectively improve visual working memory in older people with aMCI. This improvement is characterized by improved retention of information and increased processing speed. It is important to note that cognitive impairment [72] can have a negative impact on patients' ability to perform complex tasks. However, it is also an important marker of disease progression. Previous research has demonstrated the crucial role of the DLPFC in visual working memory processing [73]. Studies have also shown that functional impairment of the DLPFC is associated with abnormal visual working memory capacity [74]. Based on these findings, we propose that high‐frequency repetitive transcranial magnetic stimulation (rTMS) can potentially enhance DLPFC activity, leading to improved performance in visual working memory tasks.

### 4.7 Effects of rTMS on Attention and Executive Function

Our study showed a significant effect on the Digit Symbol Substitution Test, which demonstrated improved task accuracy and reduced processing time in individuals with aMCI and healthy older controls following rTMS treatment. Previous research has consistently shown a robust relationship between digit symbol substitution tasks and visual working memory, emphasizing the close correlation between task performance and information processing speed [75]. Several studies have suggested that, in addition to the speed of information processing, learning ability could also have an influence on performance in the Digit Symbol Substitution Test. Once a person has mastered the relationship between symbols and numbers, they no longer need to rely on visual scanning and can instead use working or episodic memory to provide correct answers. This strategy can effectively reduce the time needed to find the correct answer and consequently improve test scores. Research has shown that the decrease in processing speed is the main reason for age‐related cognitive decline [76]; improving the information processing speed of older people is therefore proving to be a key endeavor with a significant impact on delaying cognitive decline.

In addition, our study showed that the application of rTMS significantly reduced the total time to complete the Stroop_C task with interference suppression in both aMCI patients and healthy older controls. These results suggest that rTMS may improve older adults' ability to filter out irrelevant information in the Stroop_C task and improve executive function. A shorter SIE time or a higher number of correct responses indicates a more efficient interference suppression process [77]. Our study found that the number of correct responses to SIEs was significantly lower in healthy elderly controls compared to aMCI patients, suggesting that healthy elderly people are better able to suppress interfering information than aMCI patients. After rTMS treatment, there was a significant decrease in the number of correct responses to SIEs and the time required for SIEs in both aMCI patients and healthy elderly controls. However, in a pairwise comparison, rTMS significantly reduced the Stroop interference time in the healthy elderly controls, whereas there was no significant change in the aMCI group. This suggests that aMCI subjects prioritize accuracy over reaction time in the Stroop task [78].

aMCI patients experience varying degrees of decline in cognitive function with age, particularly in working memory, executive function, and processing speed [79]. Our study shows that the application of rTMS to the left DLPFC can effectively improve attention and executive functions, inhibitory control, and information processing speed in both aMCI patients and healthy older controls. In addition, a longitudinal study suggests that executive dysfunction may precede memory changes in these individuals [80]. Research on various aspects of executive function has shown that performance on the Stroop color‐word interference task can serve as a predictor of cognitive decline in older people [81]. Furthermore, our preliminary experimental study showed that rTMS did not significantly improve memory but did improve executive function. Previous studies have shown that aMCI, which is characterized by deterioration in executive function and functional status, is more likely to lead to Alzheimer's disease (AD) than memory [82]. Furthermore, our preliminary experimental study showed that rTMS did not significantly improve memory, but did improve executive function. Previous studies have suggested that aMCI, characterized by deterioration in executive function and functional status, is more likely to lead to Alzheimer's disease (AD) than memory [83]. This suggests the need to improve executive function and inhibitory control in patients with aMCI, as these factors may be critical in strengthening cognitive reserve and potentially delaying cognitive decline in this cohort.

### 4.8 Effects of rTMS on ERP Components of VWM Task

In this study, we focused on the extraction of N2pc components during the VWM coding phase. Initially, there was a significant difference in N2pc amplitude between aMCI patients and healthy controls. After 1 week of rTMS, aMCI patients showed a remarkable improvement in N2pc amplitude. This is a coherent representation of the focus of our study, the baseline findings, and the effects of rTMS on N2pc [84]. The N2pc component reflects not only the processing of the target stimulus but also the inhibition of interfering stimuli. Previous studies have shown that the N2pc component serves as a reliable marker for the allocation of attention to relevant information in visual space. This is because there is a correlation between N2pc and attentional processes during VWM encoding [33, 85]. Recent evidence suggests that the N2pc component primarily reflects the selective enhancement of cortical representations of focused objects rather than the inhibition of unfocussed objects [86]. In VWM encoding, focusing attention on the target improves the precision of memory representation. Although research on VWM encoding is limited compared to maintenance, optimizing signal processing during encoding is crucial. Repetitive transcranial magnetic stimulation (rTMS) can influence primary visual information processing, potentially increasing attention, improving control and accuracy of encoding, and enhancing visual working memory.

### 4.9 Effects of rTMS on Neuronal Oscillation of VWM Task

ERP components capture neural processing by measuring changes in the amplitude of electrical signals recorded from the scalp, which provide a comprehensive picture of the activity of many neurons. During this process, neurons in the brain undergo changes in neuronal oscillations in different frequency bands. These neuronal oscillations at different frequencies form the basis for the maintenance of VWM and are associated with various cognitive processes involved in VWM [87]. The aim of our study was to investigate the involvement of neural oscillations in different phases of VWM tasks, focusing on the encoding phase of VWM processing. We also wanted to investigate the effects of rTMS on neuronal oscillations in different frequency bands.

The alpha band is a prominent cortical rhythm observed in the posterior region of the brain. It is commonly associated with the priming effect that occurs before the presentation of a visual stimulus [88, 89, 90]. Several studies have shown that areas that are activated during a task exhibit ERD [91, 92]. Alpha activity has been found to play a crucial role in attention by supporting processes that promote concentration while inhibiting processes that lie outside the realm of attention [93]. Research repeatedly emphasizes the important role of alpha oscillations in visual processing in the posterior brain region, where alpha inhibition indicates increased neuronal excitability, and increased oscillations indicate decreased excitability [48].

These oscillations efficiently modulate cortical excitability top‐down, facilitating responses to stimuli. Our study showed that rTMS enhanced ERS in the alpha frequency band of the parieto‐occipital region in patients with aMCI [94]. We propose that deficits in occipital alpha oscillations contribute to the attentional impairments observed in aMCI patients. By enhancing alpha oscillations, rTMS may help compensate for these deficiencies. In contrast, healthy older adults, with greater cognitive reserves, can maintain normal attentional function through a variety of compensatory mechanisms [95]. Conversely, the reduced cognitive reserve and underlying functional impairments in aMCI patients make them more responsive to the therapeutic effects of rTMS. Alpha oscillations are thought to reflect top‐down attentional control processes. The modulation of alpha amplitude is an indicator of the allocation of attentional resources [96]. Alpha oscillations are strongly associated with the allocation of attentional resources. It has also been suggested that alpha oscillations are involved in the suppression of irrelevant information [97]. Alpha oscillations are strongly associated with the allocation of attentional resources. It has also been hypothesized that alpha oscillations are involved in the suppression of irrelevant information [94]. Alpha oscillations play a crucial role in protecting new memories by suppressing additional sensory processing that could potentially impair the retention of stored information [25]. Increased alpha is also thought to play a positive functional role by preventing the flow of scattered information to the areas that store memory content [98]. For example, inhibition or deactivation of the occipital‐parietal region can hinder the transmission of visual information. Increased alpha oscillations are a sign of increased vigilance [99]. Based on these findings, we propose that rTMS increases alpha oscillations in the parieto‐occipital region and thus improves top‐down control functions, increases selective attention, maintains vigilance, and, as expected, strengthens visual working.

A predominant feature of EEG patterns in Alzheimer's disease is a general slowing of the EEG waveform associated with a decrease in alpha power [100, 101]. In particular, the administration of the most commonly used drug for the treatment of Alzheimer's disease, which increases cholinergic acetyl neurotransmission, led to the restoration of alpha oscillations. This was accompanied by significant improvements in cognitive functions that rely on top‐down regulation [102]. Our study found that pre‐ and post‐repetitive rTMS assessments showed increased parieto‐occipital alpha oscillations in aMCI. This enhancement correlated with improved attention, which likely contributed to improved VWM task performance. Enhanced alpha oscillations, which are known to suppress interference, were observed throughout the VWM task phases following rTMS, suggesting that they play a critical role in delaying cognitive decline and supporting cognitive rehabilitation interventions.

### 4.10 Effects of rTMS on Task‐Related Functional Connectivity of VWM

Visual working memory processing relies on different brain regions, with the frontal–parietal region serving as a crucial hub for executive and attentional functions involved in memory maintenance [103]. Our study found that during the encoding phase of VWM, there was a concomitant improvement in connectivity between the frontal and parietal regions and between the frontal and occipital regions, particularly in the θ‐band. Remarkably, the improvement in visual working memory capacity was strongly associated with the improvement in frontal–parietal connectivity. This finding suggests that the mechanism underlying this improvement may involve the integration of information across different brain regions and emphasizes the crucial role of attention in memory formation [104]. In the VWM task, we found a strong correlation between prefrontal and parietal EEG activity. In addition, prefrontal rTMS indirectly activates the parietal and occipital lobes [99]. The synchronization of theta band oscillations plays a crucial role in cognitive tasks as it regulates the top‐down effects on task‐related cortical regions [105]. Theta oscillations have been linked to memory [23] and attentional processes [106]. Furthermore, research has shown that theta oscillations play a central role in VWM by facilitating the encoding of different types of information [21] and are considered crucial for successful memory function [22, 23]. The oscillations of the theta band play a central role in the integration of different brain regions that are necessary for visual working memory. Furthermore, the synchronized activity of the theta band between the frontal and posterior parietal regions is closely linked to the successful encoding of visual working memory [93]. Improving the synchronization of theta‐band connections between the frontal and parietal regions is crucial for improving attention and memory, increasing cognitive reserve and delaying cognitive decline in patients with aMCI. Information storage requires activity in the prefrontal and parietal regions associated with behavioral responses. Functional connectivity between these two regions has increased with rTMS. rTMS affects the parietal region indirectly via the frontoparietal pathway and utilizes the neuroplasticity of the nervous system, which is reflected in the formation of synapses, the establishment of connections, expansion, and changes in network complexity [107]. While the exact mechanism of interaction between rTMS and the nervous system remains to be further explored, numerous research findings have shown that rTMS can effectively activate specific brain regions, improve frontoparietal functional connectivity, and enhance cognitive performance. Connectivity between the frontal and parietal lobes and the occipital lobe involves a considerable number of neuronal connections and information interactions that play a crucial role in various networks [108]. For example, these connections are involved in various networks, including the sensorimotor network and the central executive network. They play a crucial role in coordinating different brain regions in functions such as vigilance, attention, information processing, and resource allocation. The study by Sun et al. has shown that the frontal, parietal, and occipital lobes together form the visual‐attentional system in visual tasks and the connection between the frontal and parietal lobes is involved in information transmission through bottom‐up feedback and top‐down control [109].

The parietal lobe plays a central role in sensory integration and the transmission of information within the network. The observed weakening of the connection between the frontal and parietal lobes in aMCI patients and the strengthening of this connection after an rTMS intervention suggest that this connection can influence the function and behavior of those affected through external stimulation and self‐regulation via neuroplasticity. Enhanced connectivity is reflected in increased information interaction and improved period‐locking efficiency, which may also be associated with improved attention and task performance in patients. The work of Buschman [108] highlights the crucial role of synchronization properties in different frequency bands for achieving bottom‐up and top‐down control at the frontal–parietal junction. In addition, our study showed an increase in α‐oscillatory power in the parieto‐occipital region after RTMS, which may be associated with increased frontal lobe reactivity during tasks and improve overall information processing efficiency. The rhythmic stimulation likely modulates cortical activity, corrects pathological patterns, improves network function, and strengthens the original model. Improved connectivity indicates improved resource allocation to synapses and loops, leading to functional improvement. Although EEG data alone do not provide direct information about polarity, our functional analysis established a link between the activity of the θ‐band of the frontoparietal junction during VWM encoding and improved performance. The study demonstrated a significant improvement in VWM task performance in aMCI patients following 10 Hz rTMS in the left DLPFC region, which impacted the frontal and parieto‐occipital regions, highlighting their critical role in VWM processing and the potential of rTMS to improve VWM task performance in aMCI patients.

Patients with aMCI often exhibit diminished functional connectivity in neural networks, particularly in regions critical for memory and attention, such as the frontoparietal network [110]. rTMS can promote neuroplasticity by targeting specific brain regions, such as the left dorsolateral prefrontal cortex, thereby enhancing the functional connectivity of these networks [111]. In contrast, healthy elders generally retain well‐preserved neural function, with their brain connectivity already at a higher baseline, which may limit the scope for further enhancement through rTMS. aMCI patients often exhibit disrupted functional connectivity in neural networks, particularly those in the prefrontal cortex and parietal regions. In our study, we also noted a significant reduction in frontoparietal connectivity in aMCI patients compared to the control group. These deficits directly impact cognitive processes such as memory encoding, retrieval, and executive function [112]. rTMS can help restore or strengthen these connections, compensating for the deficits caused by early neurodegeneration. As for healthy Controls, their neural networks are relatively intact, maintaining strong connectivity and efficient information processing. While rTMS may further optimize these connections, the ceiling effect limits how much improvement can be achieved. aMCI patients derive greater benefit from rTMS because the therapy addresses underlying deficits in neural function and connectivity, compensates for reduced cognitive reserve, and targets areas with significant room for improvement. Healthy controls, while still able to experience some cognitive benefits, exhibit smaller effects due to their relatively intact brain function and higher baseline performance. These differences underscore the importance of tailoring rTMS protocols to specific populations for optimal outcomes.

## 5 Summary and Limitations

This study represents the first investigation of the cognitive neuro‐mechanisms underlying visual working memory impairment in people with aMCI. Using an extended transformation detection task, we investigated multiple dimensions including behavior, ERPs, neural oscillatory activity, and functional brain connectivity. In addition, we used rTMS as a neuro‐modulatory technique specifically targeting the DLPFC to investigate the effects of rTMS on neural processing of visual working memory signals in aMCI patients. In this study, we observed a persistent impairment of visual working memory capacity in aMCI patients. This was reflected in reduced accuracy when completing VWM tasks, which represents a significant decrease in memory capacity. Further correlation analyses showed a close relationship between the severity of visual working memory impairment and frontal–parietal connectivity. ERP studies showed a significant reduction in the N2pc component associated with VWM tasks compared to the HEC group. We hypothesize that this may be related to impaired allocation of attentional resources and a reduced ability to selectively allocate attention. In addition, α‐oscillations in the parieto‐occipital region responsible for top‐down regulation were impaired, suggesting that reduced synchronization in the frontoparietal control network may be a crucial factor in the impairment of visual working memory in aMCI patients. The results of the second part of the study showed that rTMS significantly improved visual working memory, attention, and executive functions in aMCI patients. By stimulating the DLPFC, rTMS indirectly enhanced activity in the parietal and occipital lobes, which facilitated communication between the frontal, parietal, and occipital lobes and increased the exchange of information between brain regions. rTMS likely achieved this by improving top‐down regulation through modulation of α‐oscillation, optimizing the allocation of attentional resources, improving selective attention, increasing vigilance, and sustaining attention, thereby improving visual working memory processing.

However, some limitations of this study should be noted. Firstly, due to the lack of a sham stimulus group, it was not possible to determine whether the results could be partially explained by the placebo effect. Indeed, the main aim of this study was to identify the neural mechanism of rTMS and not to demonstrate poor efficacy under real rTMS conditions compared to sham stimulation. Secondly, the generalisability of the current research findings may be limited by the relatively small sample size of aMCI patients, which warrants further investigation with larger cohorts. Furthermore, in our study, we used conventional electrode caps (F3) to stimulate the left DLPFC, which is not as precise as MRI‐guided neuro‐navigation. Although this traditional method has been shown to be reliable and reproducible in many previous studies, future research could benefit from more accurate stimulation by neuro‐navigation systems. This requires importing three‐dimensional DICOM magnetic resonance images with magnetisation preparation and gradient echo (3D‐MPRAGE) into the Visor2 system, creating a 3D model of the patient's brain and accurately marking the left DLPFC as a stimulation target. Thirdly, the diagnosis of aMCI in this study was not based on biomarkers, which limited the accuracy of the diagnosis. Traditional diagnostic criteria for aMCI primarily focus on clinical presentation, including reports of subjective memory decline, mild cognitive abnormalities, and preserved daily functioning (e.g., Petersen criteria). Which is widely used due to its simplicity and ease of implementation. As the previous study showed that people with aMCI have a significantly higher risk of developing AD, with about 10%–15% of aMCI patients progressing to AD annually. In comparison, the general population has a much lower annual incidence of AD, around 1%–2% [113]. Due to the high cost of PET‐CT scans and the risks associated with lumbar puncture procedures, many participants in the study preferred non‐invasive assessments such as cognitive evaluations and blood tests, which further limited the inclusion of biomarker data. With the advancement of plasma biomarker testing for Alzheimer's disease, the detection and diagnosis of AD have become more accessible and reliable. Future studies should incorporate biomarker assessments, such as Aβ42, p‐tau181, p‐tau217, to improve diagnostic accuracy. Exploring their correlation with disease progression in aMCI and Alzheimer's disease could provide valuable insights into neurobiological mechanisms. This may aid in early diagnosis and more accurate predictions of cognitive decline, enhancing understanding of the relationship between aMCI and its underlying mechanisms. Finally, our research focused mainly on the short‐term effects of rTMS. Future studies will improve follow‐up plans to explore the lasting effects of rTMS by conducting behavioral and EEG assessments 1 month and 3 months after completion of rTMS treatment. This will provide better evidence for clinical interventions.

## Author Contributions

**Meng Liu:** study design, data collection, statistical analysis, data interpretation, manuscript editing, and literature search. **Jingnan Sun:** study design, manuscript revision, review, and editing. **Janelle S. Y. Yeo:** data collection, manuscript translation, and literature search. **Ren Ren‐Li:** data collection, statistical analysis, and literature search. **Jing Ma:** data collection, statistical analysis, and literature search. **Jia‐Xin Yan:** data collection, editing, and literature search. **BuMaYiLaMu‐XueKeEr:** data collection, statistical analysis, and literature search. **Zhao‐Xi Tu:** data collection and literature search. **Yun‐Xia Li:** study design, supervision, manuscript revision, fundraising. All authors contributed to the article and approved the submitted version.

## Ethics Statement

The study was approved by the Ethics Committee of Tongji Hospital (No. (Tong) ethical review 429), and all subjects signed an informed consent form.

## Conflicts of Interest

The authors declare no conflicts of interest.

## Supporting information


Data S1.

## Data Availability

The data supporting the conclusions of this article are available from the corresponding author upon reasonable request.
